# Characterization of Long-period Grating Refractive Index Sensors and Their Applications

**DOI:** 10.3390/s90604559

**Published:** 2009-06-10

**Authors:** Hiroshi Tsuda, Kei Urabe

**Affiliations:** Research Institute of Instrumentation Frontier, National Institute of Advanced Industrial Science & Technology, Tsukuba Central 2, Tsukuba 305-8568, Japan; E-Mail: urabe-k@aist.go.jp

**Keywords:** long-period grating, refractive index, spectral sensitivity

## Abstract

The influence of grating length and bend radius of long-period gratings (LPGs) on refractive index sensing was examined. Sensitivity to refractive indexes smaller than that of silica could be enhanced by bending LPGs. Bent LPGs lost sensitivity to refractive indexes higher than that of silica, whereas a 20-mm-long LPG arranged in a straight line had considerable sensitivity. These experimental results demonstrated that the sensitivity characteristics of LPGs to refractive index could be controlled by grating length and bend radius.

## 1. Introduction

The measurement of refractive index is important to many fields such as bio-chemical analysis, environmental contamination assessment, and the food and chemical industries. Because of the wide range of applications mentioned above, there is a growing interest in optical refractive index sensors. Those based on photonic crystal fibers (PCFs) or fiber gratings are probably the most attractive ones since they offer high sensitivity, stable wavelength-modulated information, and broad measuring range. At a refractive index of 1.33 typical for aqueous environments, the sensitivity of a fiber grating-based sensor is typically 50 nm/refractive index units (RIU) [1], while PCF-based sensors possess higher sensitivity of more than 1,500 nm/RIU [2,3]. However, PCF-based sensors have the drawback of cumbersome measurement procedures. In the measurement of refractive index, the tiny holes of PCF must be filled with the liquid to be measured and the infiltration of liquid typically takes at least several minutes. Furthermore, the liquid filling the holes must be eliminated for the subsequent measurement. On the other hand, fiber grating-based sensors are capable of responding to refractive index change immediately and are thus suitable for continuous real-time monitoring.

Fiber gratings are often classified as fiber Bragg gratings (FBGs) or long-period gratings (LPGs), according to grating period. LPGs typically have a grating period in the range of from 100 μm to 1 mm, whereas FBGs have a sub-micron period. The transmission spectrum of LPGs consists of a number of rejection bands that arise from light coupling of the fundamental core mode to the multiple cladding modes. A central wavelength of rejection band *λ_rej_* of an LPG with grating period Λ is given by the phase-matching condition:

(1) $$\lambda_{\text{rej}}=({n_{\text{core}}}^{\text{eff}}-{n_{\text{cladding},m}}^{\text{eff}})\Lambda$$

where *n_core_^eff^* and *n_cladding,m_^eff^* are the effective refractive indexes of the fundamental core mode and the m*th* cladding mode, respectively. Changes in the temperature, strain, and refractive index of the medium surrounding the LPG can alter the grating period and/or the differential refractive index of the core and cladding modes. Thus, the rejection wavelengths of LPG are sensitive to such environmental changes [4].

Applications of fiber gratings to sensors for strain and temperature measurement have been studied over the last two decades [5-9]. Fiber Bragg gratings have been widely applied to strain and temperature sensors because they have a narrow reflection band, which contributes to higher resolution. Iadicicco *et al.* have reported refractive index (RI) measurement using FBGs, in which the cladding of the grating section was thinned by etching in order to possess RI sensitivity [10]. The FBG RI sensor lacks robustness because an FBG with a thin cladding breaks easily. On the other hand, an LPG with no protective coating has RI sensitivity because its *n_cladding,m_^eff^* is affected by the RI of the surrounding medium. Long-period gratings can be simple and robust RI sensors compared with FBGs, and so a number of studies on the RI sensitivity of LPGs have been conducted [11-17].

A previous study on RI sensing using LPGs by Khaliq *et al.* has demonstrated that a change in external RI causes not only a shift in the rejection band wavelength but also a change in the transmission of the rejection band [18]. According to their study, the spectral change of LPG RI sensors can be characterized in terms of external RI as follows. For an external RI lower than that of silica, the rejection band shifts to a lower wavelength with increasing RI, while its transmission changed insignificantly. At an external RI equal to that of silica, rejection bands disappeared, and the transmission spectrum flattened. For an RI higher than that of silica, the rejection bands reappeared, but with high transmission and no wavelength response to external RI change.

Refractive index sensing with LPGs uses light coupling between core and cladding modes in the grating section. The grating length and bend radius of LPGs are thought to influence the light coupling significantly. However, the effect of grating length and bend radius on the RI sensitivity remains unclear. In the present study, the influence of the grating length and bend radius of LPG on transmission spectra at various external RIs was examined. We found a suitable configuration of LPG for RI sensing, which could be classified according to external RI. Then, LPG RI sensors were used in two applications: a discrimination test between water and saline water having an RI lower than that of silica and cure monitoring of epoxy resin for which the RI was higher than that of silica.

## 2. Experimental Procedure

Two LPGs with grating lengths of 20 mm and 30 mm, respectively, were used, and both LPGs had grating periods of 450 μm. There was no protective coating in the grating section, so that the external RI could affect the effective refractive index of cladding modes. We measured the transmission spectra for the cases in which the LPG was surrounded by air, water, or one of four standard RI liquids (Cargille Laboratories, USA) in order to examine the RI sensitivity of the LPG. The refractive indexes of these four liquids were 1.4, 1.45, 1.5, and 1.6 at 1,550 nm. Furthermore, in order to investigate the bending effect on RI sensitivity, we examined the spectral difference between a straight LPG and a bent LPG at various external RIs.

## 3. Results and Discussion

### 3.1. Influence of grating length on RI sensitivity

**Figure 1.**
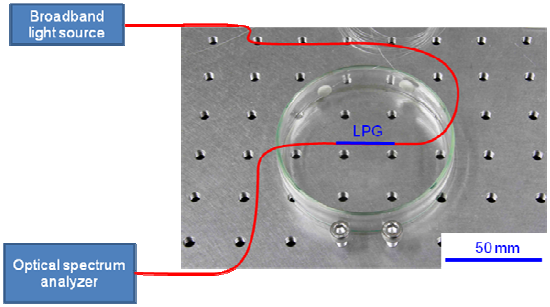
Experimental setup for measuring the transmission spectrum of a straight LPG.

**Figure 2.**
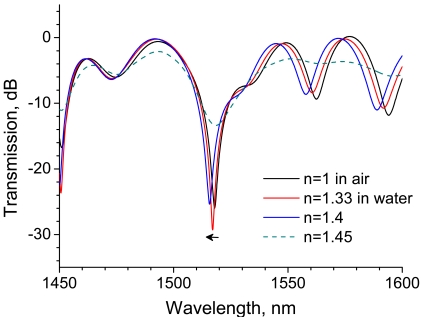
Transmission spectra of a 30-mm-long straight LPG surrounded by a medium having a refractive index that is lower than that of silica.

We first investigated the RI sensitivity of a 30-mm-long LPG, which was arranged in a straight line on a Petri dish, as shown in Figure 1. A super-luminescent diode, the output wavelength of which ranged from 1,300 to 1,600 nm, was used as a light source. Figure 2 shows the transmission spectra of the LPG surrounded by a medium having an RI that is lower than that of silica. A strong rejection band having a central wavelength around 1,520 nm is observed, and the band shifts to a shorter wavelength by 2.3 nm as the RI increases from 1 to 1.4. The spectrum begins to flatten out at an external RI of 1.45, which is close to the RI of 1.456 of silica.

**Figure 3.**
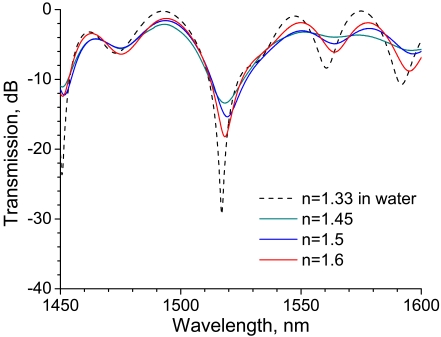
Transmission spectra of a 30-mm-long straight LPG surrounded by a medium having a refractive index that is higher than that of silica.

The transmission spectra of the LPG surrounded by a medium having an RI higher than that of silica is shown in Figure 3 with a reference spectrum at an RI of 1.33, where the LPG was immersed in water. Note the rejection band around 1,520 nm. Compared with the reference spectrum, the transmission of the rejection band increases. Among the spectra for which the external RI is higher than that of silica, the transmission of the rejection band decreases with RI. The spectral features observed in Figures 2 and 3 are consistent with those reported previously [11-13,17,18].

**Figure 4.**
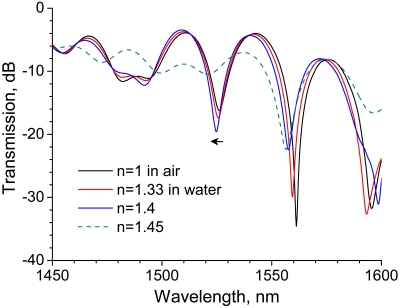
Transmission spectra of a 20-mm-long straight LPG surrounded by a medium having a refractive index that is lower than that of silica.

As mentioned in the previous section, the grating length of the LPG influences the coupling effect between the core and cladding modes. In order to investigate the influence of grating length on RI sensitivity, we performed the same experiment using a 20-mm-long LPG. Figure 4 shows transmission spectra of a 20-mm-long LPG surrounded by a medium having an RI lower than that of silica. Similarly to the case of 30-mm-long LPG shown in Figure 2 the spectra have a rejection band around 1,520 nm, while a rejection band having higher attenuation appears at a longer wavelength (around 1,560 nm). Note the wavelength shift in the rejection band around 1,520 nm which is the same rejection band we noted in Figures 2 and 3. The wavelength is shifted by 1.4 nm as external RI increases from 1 to 1.4, whereas the shift was 2.3 nm in the case of the 30-mm-long LPG. These experiments revealed that longer LPGs had higher sensitivity to RIs lower than that of silica.

**Figure 5.**
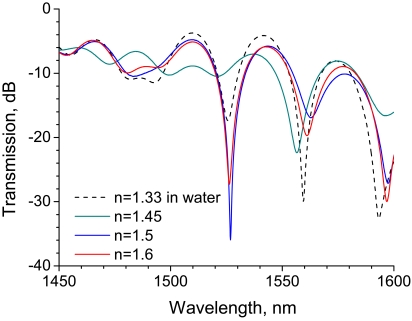
Transmission spectra of a 20-mm-long straight LPG surrounded by a medium having a refractive index that is higher than that of silica.

The transmission spectra of a 20-mm-long LPG surrounded by a medium having an RI that is higher than that of silica are shown in Figure 5, along with the reference spectrum at an RI of 1.33. Compared with the reference spectrum, the transmission of the rejection band around 1,520 nm decreases considerably. Furthermore, the transmission increases with external RI as long as the RI is higher than that of silica. These features are inconsistent with the present experimental results for a 30-mm-long LPG shown in Figure 3 as well as those reported in previous studies [12,13,18]. All of the LPGs used in the previous studies had a grating length of over 25 mm. The reduction in grating length might have given rise to the anomalous spectral response to an RI higher than that of silica.

### 3.2. Effect of bending on RI sensitivity

**Figure 6.**
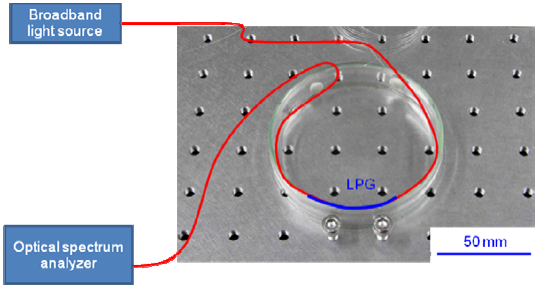
Experimental setup for measuring the transmission spectrum of a bent LPG.

The bend of the LPG would also affect the coupling between the core and cladding modes. To investigate the effect of bending on the RI sensitivity, a 20-mm-long LPG was arranged along the inner wall of a Petri dish having a bend radius of 43 mm, as shown in Figure 6, and the transmission spectra at various external RIs were recorded.

**Figure 7.**
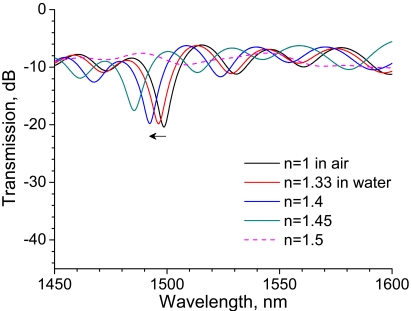
Transmission spectra of a 20-mm-long bent LPG surrounded by a medium having a refractive index ranging from 1 to 1.5.

The experimental results are shown in Figure 7. Compared with spectra of the straight LPG shown in Figure 4, the depth of the rejection band is shallow and the strongest rejection band shifts to a shorter wavelength (around 1,500 nm). The rejection peak shifts by 6.3 nm as the RI is increased from 1 to 1.4, which is 4.5 times longer than the wavelength shift for the straight LPG.

In addition, the transmission spectrum flattens out at an external RI of 1.5. The transmission characteristics are quite different from those of the straight LPG shown in Figure 5. The bend of the LPG would promote the leaky loss of cladding modes, and thus there would be little coupling between the core and cladding modes in the bent LPG. As a result, the bent LPG would have had an almost flat transmission spectrum. These experimental results indicate that bent LPGs are suitable for monitoring RIs lower than that of silica, but are inappropriate for monitoring RIs higher than that of silica.

### 3.3. Discrimination between water and saline water using a bent LPG

**Figure 8.**
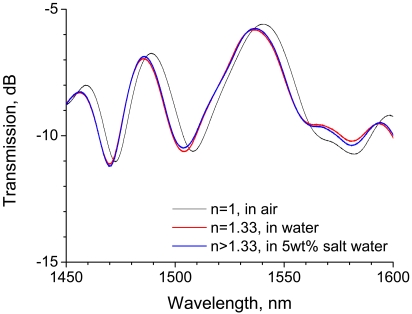
Discrimination of water from saline water using a 20-mm-long LPG having a bend radius of 43 mm.

The RI of water is 1.33, which is lower than that of silica (1.456), and saline water has a higher RI than water. In the present study, we applied a bent LPG, which is suitable for monitoring RIs lower than that of silica, to discriminate between water and 5 wt% saline water. The experimental setup shown in Figure 6 was used in this test. The transmission spectra of the 20-mm-long LPG with a bend radius of 43 mm are shown in Figure 8. Compared with the spectrum at an RI of 1, where LPG was surrounded by air, the spectra of the LPGs immersed in water and saline water shift to shorter wavelengths. However, the LPG with a bend radius of 43 mm lacks the RI sensitivity needed to discriminate between water and 5 wt% saline water because the spectral shift between water and 5 wt% saline water is scarcely distinguishable.

**Figure 9.**
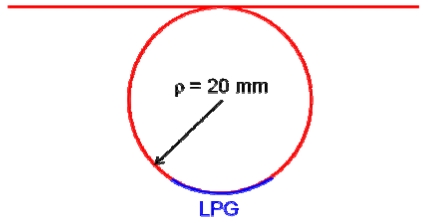
Schematic diagram of a 20-mm-long LPG having a bend radius of 20 mm.

**Figure 10.**
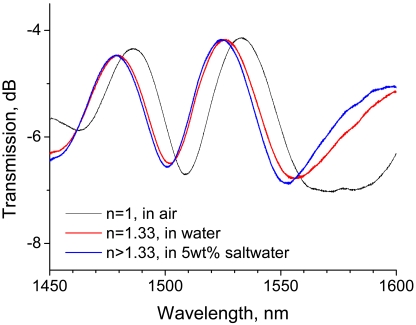
Discrimination of water from saline water with a 20-mm-long LPG having a bend radius of 20 mm.

The authors conjectured that an LPG having a smaller bend radius would have higher RI sensitivity. Hence, we devised a 20-mm-long LPG with a bend radius of 20 mm, as depicted in Figure 9, and performed the discrimination test. Figure 10 shows the resulting spectra. A 1.6-nm wavelength shift at the rejection band around 1,500 nm can be observed between the two spectra for which the LPG was immersed in water and saline water. These tests reveal that bent LPGs with smaller curvatures have higher sensitivity to external RIs that are lower than that of silica.

### 3.4. Epoxy resin cure monitoring using an LPG

**Figure 11.**
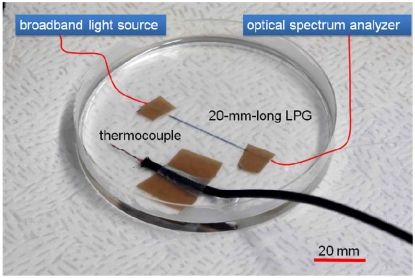
Experimental setup for monitoring epoxy resin cure.

Monitoring of the resin cure process is important in order to secure the quality and reliability of polymer and composite materials. Dielectric characterization is one of the methods conventionally applied to monitor cure process of polymers [19]. Since the RI is closely related to the dielectric constant, RI sensing with LPGs is expected to be an effective method by which to monitor the cure process. In the present study, the cure process of epoxy resin was monitored using an LPG by measuring the transmission spectrum. The base resin and hardening agent of the epoxy were Epikote 828 (Japan Epoxy Resins Co., Ltd., Japan) and diethylenetriamine, respectively. According to the provider, the cured epoxy resin has a refractive index in the range of from 1.55 to 1.6 [20]. Since the RI of epoxy is higher than that of silica, a straight LPG suitable for the monitoring of higher RI was applied to the cure monitoring. As shown in Figure 11, we arranged a 20-mm-long LPG in a straight line along with a thermocouple for temperature measurement.

**Figure 12.**
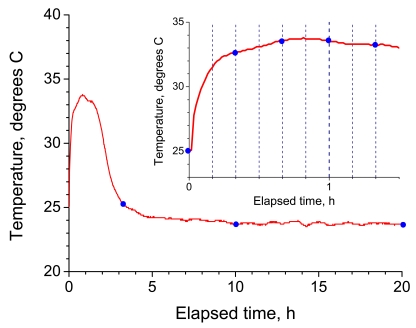
Temperature profile in the epoxy resin cure process. The upper right figure is the enlarged temperature profile until 90 minutes after pouring the resin. The blue circles indicate the times at which the transmission spectra were recorded.

Figure 12 shows the temperature change during the cure process. The temperature soared just after the epoxy resin was poured and reached a plateau after 40 to 80 minutes before gradually declining. The epoxy resin started to become viscous after 1 hour, at which time the temperature reached a maximum. The epoxy resin was almost cured after 3 hours, at which time the temperature was close to that of the ambient atmosphere. The circular symbols in Figure 12 indicate the points at which the transmission spectrum of the LPG immersed in epoxy resin was recorded.

**Figure 13.**
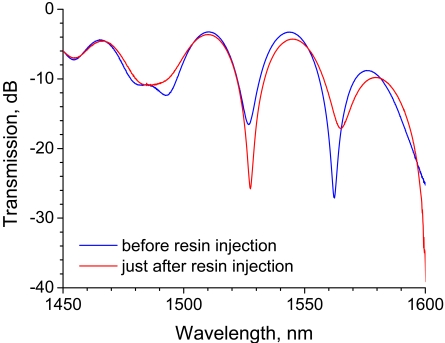
Change in transmission spectra of an LPG before and after resin infusion.

The transmission spectra before and after resin injection are shown in Figure 13. Similarly to the experimental results shown in Figure 5, strong rejection bands appear in the spectra around 1,525 nm. The transmission of the rejection band decreases after resin injection. This change in the spectrum is consistent with the experimental results shown in Figure 5, in which the transmission of the rejection band around 1,525 nm decreased when the 20-mm-long LPG was surrounded by a medium having an RI that is higher than that of silica. Since the transmission of the rejection band decreased significantly after the LPG was surrounded by resin, the LPG can act as a resin flow-front sensor.

**Figure 14.**
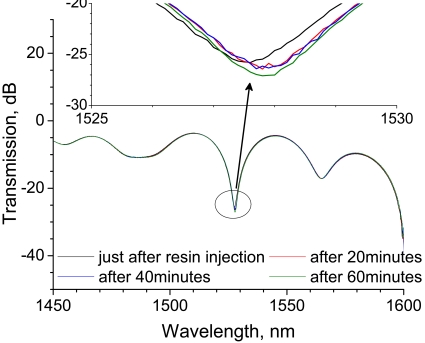
Change in the transmission spectra of an LPG in the resin cure process in which the temperature was saturated. The upper figure is an enlarged view of the transmission spectra in the wavelength range from 1,525 to 1,530 nm.

Figure 14 shows the LPG spectral change for 60 minutes after resin injection. At a glance, the spectral change appears to be insignificant for the first 60 minutes of the cure process. However, the enlarged view of the spectra at around 1,525 nm shows that transmission of the band after 60 minutes decreases by 1.4 dB, as compared with the spectrum just after resin injection. Furthermore, the band shifts to a longer wavelength after 20 minutes and remains constant until 60 minutes. The band shift to a longer wavelength is thought to correspond to temperature increase.

**Figure 15.**
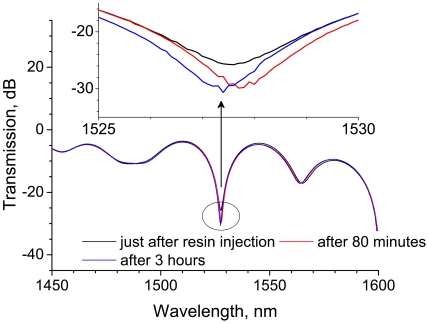
Change in transmission spectra of an LPG in the resin cure process in which temperature decreased gradually. The upper figure is an enlarged view of the transmission spectra in the wavelength range from 1,525 to 1,530 nm.

**Figure 16.**
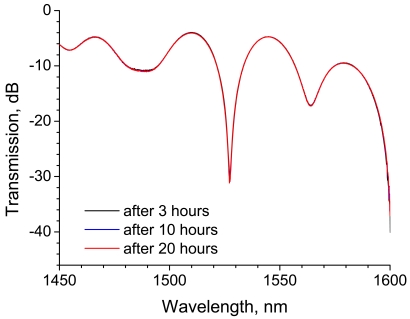
Transmission spectra of an LPG after resin cure.

The LPG spectra after 80 minutes and after 3 hours are shown in Figure 15. The transmission of the rejection band decreases by 4.3 dB after 80 minutes and it remains approximately constant until 3 hours, but the central wavelength shifts to a shorter wavelength. As mentioned above, the return of the central wavelength is thought to correspond to the temperature decrease. Figure 16 shows the spectra obtained after 3, 10, and 20 hours. There is no discernible change in these spectra.

The influence of RI on the spectral change of the straight 20-mm-long LPG is shown in Figure 5, and similar spectral behavior is expected for the cure monitoring. The experimental results shown in Figure 5 demonstrated that the transmission of the rejection band around 1,525 nm at RIs of 1.5 and 1.6 were -36.0 and -27.3 dB, respectively, and the transmission at the rejection band increased with external RI. In the cure monitoring test, the transmission at the rejection band changed from -25.7 to -31.1 dB, and the transmission decreased with the curing progress. The following can be inferred from the comparison of the spectral change in these two cases. The RI of the resin just after injection must have been greater than 1.6 because the transmission just after resin injection (-25.7 dB) was higher than that for which the external RI was 1.6 (-27.3dB). Then, the RI would have decreased with the progress of curing because the transmission decreased with time. This behavior in which the RI decreased with progress of curing is consistent with the results of a previous study on cure monitoring of the resin with conventional dielectric measurement [21]. The cured resin would have had an RI in a range of 1.5 to 1.6 because the transmission of cured resin (-31.1 dB) was within the range of -36.0 to -27.3 dB, the corresponding RIs of which were 1.5 and 1.6, respectively. The RI estimated from the spectral change of the LPG is in good agreement with the typical RI of epoxy resin (1.55 to 1.6).

## 4. Conclusions

The influence of grating length and bend radius of LPGs on RI sensitivity was investigated. Long-period gratings responded to RI variation by a wavelength shift in the transmission spectrum when the RI to be monitored was lower than that of silica. Compared with LPGs arranged in a straight line, bent LPGs had higher sensitivity, and the sensitivity could be improved by reducing bend radius. On the other hand, when the RI to be monitored was higher than that of silica, the bend of the LPG eliminated the rejection bands, and thus bent LPGs had no RI sensitivity. A straight 20-mm-long LPG demonstrated a considerable change in spectral transmission in response to an RI shift from 1.5 to 1.6.

Long-period gratings were applied to two applications: water quality examination and resin cure monitoring, in which the RIs were lower and higher than that of silica, respectively. An LPG with a bend radius of 20 mm could discriminate between water and 5 wt% saline water from the transmission spectral shift. The epoxy resin cure process was monitored using a straight 20-mm-long LPG. The LPG detected changes in the RI during the cure process as the shift in the transmission level of the rejection band.

## References

[1] Chong J.H., Shum P., Haryono H., Yohana A., Rao M.K., Lu C., Zhu Y.N. (2004). Measurements of refractive index sensitivity using long-period grating refractometer. Opt. Commun..

[2] Rindorf L., Bang O. (2008). Highly sensitive refractometer with a photonic-crystal-fiber long-period grating. Optics Letters.

[3] Wu D.K.C., Kuhlmey B.T., Eggleton B.J. (2009). Ultrasensitive photonic crystal fiber refractive index sensor. Optics Letters.

[4] James S.W., Tatam R.P. (2003). Optical fibre long-period grating sensors: Characteristics and application. Meas. Sci. Technol..

[5] Melle S.M., Liu K.X., Measures R.M. (1993). Practical fiberoptic Bragg grating strain-gauge system. Appl. Opt..

[6] Othonos A. (1997). Fiber Bragg gratings. Rev. Sci. Instrum..

[7] Lee B.H., Nishii J.J. (1998). Bending sensitivity of in-series long-period fiber gratings. Optics Letters.

[8] Jung J., Nam H., Lee B., Byun J.O., Kim N.S. (1999). Fiber Bragg grating temperature sensor with controllable sensitivity. Appl. Opt..

[9] Han Y.G., Lee B.H., Han W.T., Paek U.C., Chung Y. (2000). Fibre-optic sensing applications of a pair of long-period fibre gratings.

[10] Iadicicco A., Cusano A., Cutolo A., Bernini R., Giordano M. (2004). Thinned fiber Bragg gratings as high sensitivity refractive index sensor. IEEE Photonic Technol. Lett..

[11] Bhatia V., Vengsarkar A.M. (1996). Optical fiber long-period grating sensors. Optics Letters.

[12] Lee B.H., Liu Y., Lee S.B., Choi S.S., Jang J.N. (1997). Displacements of the resonant peaks of a long-period fiber grating induced by a change of ambient refractive index. Optics Letters.

[13] Duhem O., Henninot J.F., Warenghem M., Douay M. (1998). Demonstration of long-period-grating efficient couplings with an external medium of a refractive index higher than that of silica. Appl. Opt..

[14] Duhem O., Henninot J.F., Douay M. (2000). Study of in fiber Mach-Zehnder interferometer based on two spaced 3-dB long period gratings surrounded by a refractive index higher than that of silica. Opt. Commun..

[15] Ng M.N., Chen Z.H., Chiang K.S. (2002). Temperature compensation of long-period fiber grating for refractive-index sensing with bending effect. IEEE Photonic Technol. Lett..

[16] Shu X.W., Zhang L., Bennion I. (2002). Sensitivity characteristics of long-period fiber gratings. J. Lightwave Technol..

[17] Tang J.L., Wang J.N. (2008). Chemical sensing sensitivity of long-period grating sensor enhanced by colloidal gold nanoparticles. Sensors.

[18] Khaliq S., James S.W., Tatam R.P. (2002). Enhanced sensitivity fibre optic long period grating temperature sensor. Meas. Sci. Technol..

[19] Levita G., Livi A., Rolla P.A., Culicchi C. (1996). Dielectric monitoring of epoxy cure. J. Polym. Sci., Part B: Polym. Phys..

[20] Available online: http://www.rontec.co.jp/basic/plastic/charaep.htm

[21] Urabe K., Takahashi J., Tsuda H., Kemmochi K. (2000). Cure monitoring of matrix resin with high-frequency electromagnetic wave transmission line. J. Reinf. Plast. Compos..

